# Combinational Radiotherapies Improve Brain Cancer Treatment at High Dose Rates In Vitro

**DOI:** 10.3390/cancers17101713

**Published:** 2025-05-20

**Authors:** Michael Valceski, Elette Engels, Sarah Vogel, Jason Paino, Dylan Potter, Carolyn Hollis, Abass Khochaiche, Micah Barnes, Alice O’Keefe, Matthew Cameron, Kiarn Roughley, Anatoly Rosenfeld, Michael Lerch, Stéphanie Corde, Moeava Tehei

**Affiliations:** 1 Centre for Medical Radiation Physics, University of Wollongong, Wollongong, NSW 2522, Australia; 2 Building 42 Molecular Horizons, University of Wollongong, Wollongong, NSW 2522, Australia; 3 Australian Synchrotron, Australian Nuclear Science and Technology Organisation (ANSTO), 800 Blackburn Road, Clayton, VIC 3168, Australia; 4 Prince of Wales Hospital, Randwick, NSW 2031, Australia

**Keywords:** synchrotron, radiosensitiser, chemotherapy, high-Z, radiotherapy, DNA damage, localisation, multi-modal, halogenated pyrimidine, drugs

## Abstract

Background/objectives: Brain cancer remains difficult to treat, with survival statistics stagnant for decades. The resistance of glioblastoma brain tumours can greatly challenge the effectiveness of conventional cancer radiotherapy. However, high dose rate radiotherapy has unique effects that allow for normal tissue sparing whilst maintaining tumour control. The addition of targeted radiosensitisers, such as the chemotherapeutic drug methotrexate (MTX) or the high-Z halogenated pyrimidine drug iododeoxyuridine (IUdR), can improve radiotherapy outcomes. Combining these radiosensitiser agents with ultra-high dose rate (UHDR) synchrotron X-rays can bear synergistic effects to enhance the efficacy of these multi-modal UHDR therapies, providing a means to overcome the radioresistance of brain cancer. Methods: Here, we use controlled in vitro assays following treatment, including a clonogenic assay to determine long-term cell survival and γH2AX immunofluorescent confocal microscopy to quantify double-strand DNA breaks (DSBs). Results: We find significant enhancement for highly synergistic combinations of IUdR+MTX with synchrotron X-rays. Cell survival results demonstrate 5.4 times increased 9L gliosarcoma cell killing when these agents are combined with UHDR synchrotron X-rays compared with conventional X-rays alone at the same 5 Gy dose. The underlying mechanisms are unveiled using γH2AX imaging and reveal significant increases in DSBs and dying cells following exposure to UHDR radiation. Conclusions: Our results demonstrate that highly synergistic combination treatments using UHDR synchrotron radiation can yield significantly improved brain cancer killing compared with conventional radiotherapy. We anticipate that these additive, multi-modal combination therapies will provide options for more targeted and effective use of radiotherapies for the future treatment of brain cancer.

## Simple Summary

Brain cancer is notoriously resistant to conventional treatments. New and improved methods have long been needed to improve outcomes. This has included the use of radiosensitisers combined with chemotherapy drugs to enhance conventional radiotherapies (RTs) and novel techniques. This includes ultra-high dose rate (UHDR) RT, which has been known to spare normal tissue whilst retaining tumour control. Using a clonogenic cell survival assay and γH2AX confocal imaging, we show a significant increase in cancer killing and DNA damage in 9L gliosarcoma brain cancer cells using UHDR X-rays (produced by synchrotron light sources). These dose rate effects demonstrate significant additive effects with high-Z iododeoxyuridine radiosensitisers combined with methotrexate drugs. Notable increases in DNA damage and possible cell death were observed following combinational treatment with these drugs and UHDR synchrotron X-ray fields. While UHDR effects are normally associated with tissue sparing, our results show an increased cancer-killing effect even with highly resistant 9L cells. Particularly, we show significant synergy when drugs are used to enhance the UHDR fields. This suggests a potential and interesting new option for using high dose rates and combinational RTs to improve brain cancer treatment.

## 1. Introduction

Cancer is a major cause of death, with an estimated 7.6 million deaths each year globally [1]. Glioblastoma multiforme (GBM) is a primary brain glioma, having among both the lowest and most stagnant 5-year survival rates and some of the highest incidence among these types of cancers [2,3].

The conventional methods of surgical removal, chemotherapy, and radiotherapy remain the common modalities for cancer treatment, although each face their own limitations [4,5,6]. Brain cancers can be difficult, if not impossible, to safely surgically resect due to their depth in the brain, whilst chemotherapeutic drugs can affect normal tissues in the body and can face difficulty in crossing the blood–brain barrier (BBB) [7,8,9]. External radiation treatments use ionising radiation (IR) to more geometrically target and damage tumours. The damage induced in cancer cells occurs either directly or indirectly through events including ionisation of target biomolecules (e.g., DNA), free radical activity resulting from radiolysis of water, and even production of secondary electron radiation, which can all produce genetic lesions [5,7,8,10].

The two primary modes of DNA damages following exogenous IR are single-strand DNA breaks (SSBs), where just one strand of the DNA double helix is discontinued and which comprise most of the breaks, and DNA double-strand breaks (DSBs), where both strands are severed and the lesion is the most lethal [11,12,13,14]. However, radiotherapy (RT) also faces the challenge of maintaining tumour control whilst limiting exposure to adjacent healthy tissues, which can experience the same DNA damage [8,10]. As radiation doses are then restricted by the tolerance of normal tissues to exposure, new and improved methods of targeted radiation treatment are needed to widen the therapeutic window.

One option is FLASH-RT, which involves the ultra-fast delivery of therapeutic radiation doses, often at rates orders of magnitude above conventional clinical modalities [15,16,17,18]. FLASH effects have been observed with radiation delivered at average dose rates ≥ 40 Gy/s [15,16,17,18]. The high dose rate (HDR) effects seen with FLASH-RT have the benefit of protecting healthy tissues whilst maintaining tumour control [15,16,17,18,19].

Similarly, HDR kilovoltage X-rays generated by synchrotron radiation sources have been observed to induce tumour control and at intrinsic dose rates as high as hundreds or even thousands of Gy/s, well within the range of potential FLASH-RT effects [15,18,19,20,21,22]. The work of Engels et al. shows the effect of ultra-HDR (UHDR) synchrotron broadbeam fields (SBB), as well as synchrotron microbeam collimations, where a notable dose rate effect is observed to significantly reduce 9LGS cell survival [19].

Besides UHDR SBB, which may provide a novel RT modality to control cancer whilst protecting normal cells, other possibilities exist. One option is combining RT with radiosensitiser agents. Radiosensitisers are enhancing agents that increase local radiation dose in targeted areas, thereby improving RT damage to tumours [23,24,25,26]. Conversely, these agents, including nanoparticles and high-Z drugs, could further permit a lower radiation dose to be used whilst maintaining RT efficacy via radiosensitisation.

Halogenated pyrimidine drugs are one such high-Z example. These agents are nucleoside analogues bound to halogen elements, making them useful anti-cancer drugs due to their disruption and replacement of thymidine DNA bases [27,28]. High-Z halogens can then deliver additive effects in combination with RT, including iodine (I, Z = 53) halogens forming iododeoxyuridine (IUdR). These DNA-localised radiosensitisers provide an opportunity for significant dose enhancement to cancer due to their proximity to DNA, whereby secondary election production from these high-Z halogens has the potential to induce localised DNA damage [26,29,30,31].

For drugs such as IUdR, exposure at 10 µmol/L (10 µM) for two cell doubling times has previously shown notable effect. 9LGS cells treated with bromodeoxyuridine (BrUdR), a bromine-based equivalent to IUdR, have seen significantly reduced survival after 125 kVp conventional broadbeam (CBB) irradiation [30,31]. Fujii et al., for the same 10 μmol/L concentration of BrUdR, observed dose enhancement and increased DSB induction on Chinese hamster ovary (CHO10B2) cells [32]. Corde et al. observed a significant enhancement with IUdR using synchrotron X-rays, where UHDR SBB fields produced a sensitisation enhancement ratio (SER) at 10% clonogenic cell survival (SER_10%_) as high as 2.5 with SQ20B squamous carcinoma cells pre-treated with 10 μmol/L of IUdR [29]. Considering the higher Z of I compared to Br (Z = 35) and the significant enhancement of IUdR with both CBB and SBB RT modalities, IUdR is a desirable candidate for continued investigation given that significant cell-killing potential may be needed to overcome the notorious resistance of GBM.

Another chemotherapeutic option is methotrexate (MTX), a well-established anti-cancer drug. MTX is a folate analogue that disrupts enzymes such as dihydrofolate reductase (DHFR), which are involved in tetrahydrofolate synthesis. This then inhibits the production of folate, which in turn disrupts cellular proliferation and DNA synthesis and repair [31,33]. Folate surface receptors are generally over-expressed on cancer cells, including brain cancer, permitting an affinity for fast-cycling tumour cells over normal tissues [34,35]. These characteristics make MTX a useful candidate for radiosensitising cancer cells to RT, whereby the cell response to radiation damage will be suppressed, including regrowth and DNA repair [33].

With multiple radiosensitiser options available, these agents may be combined to produce additive effects. Combinational therapies have been found previously to synergistically improve treatment efficacy, including combinations of chemotherapeutic drugs, nanoparticles, RT, and even internal radionuclides [36,37,38]. Accordingly, MTX can be combined with other agents to obtain these additive effects, allowing other radiosensitisers, such as IUdR, to synergistically amplify dose enhancement and cancer survival reduction even further.

This has been observed with combinations of BrUdR and MTX, another chemotherapeutic antimetabolite, where MTX has produced supra-additive effects beyond the enhancement provided by either agent alone [30,31]. Considering the affinity of MTX for tumours and the normal-tissue-sparing effects of novel RT methods like UHDR SBB, these findings present an excellent opportunity for combining multiple treatment modalities for a highly synergistic effect. A multi-modal combination of high-Z radiosensitisers with chemotherapeutic agents and UHDR synchrotron X-rays may provide a supra-additive means of targeted tumour control whilst sparing normal tissues.

Accordingly, in this study, we seek to build upon our previous work in this area [39] and investigate the combinational effects of IUdR and MTX with conventional (CBB) or UHDR synchrotron broadbeam (SBB) irradiation in vitro, thereby comparing conventional dose rates (CDR) with UHDR X-rays. Our biological endpoints are clonogenic assays and γH2AX DSB imaging by confocal microscopy on the 9LGS cell line, a widely used rodent cell model for radioresistant gliosarcoma and GBM cancers more generally [1,10,40,41,42].

## 2. Materials and Methods

### 2.1. Subculture of Adherent Cells

We obtained 9L gliosarcoma (9LGS) cells from the European Collection of Authenticated Cell Cultures (ECACC). 9LGS cells were cultured in T75 cm^2^ flasks (Greiner Bio-One via Interpath, Melbourne, VIC, Australia, #658175) containing complete Dulbecco’s Modified Eagle Medium (c-DMEM) (Gibco, Brisbane, QLD, Australia, #11965118), with 10% Foetal Bovine Serum (FBS) (Gibco via ThermoFisher Scientific, Brisbane, QLD, Australia, #10099141) and 1% PenStrep (10,000 units/mL penicillin, 10,000 μg/mL streptomycin) (Gibco via ThermoFisher Scientific, Brisbane, QLD, Australia #15140122). Cultures were incubated at 37 °C and 5% (*v*/*v*) CO_2_, and all 9LGS cells had a doubling time of 36 h.

When harvested or passaged, cells were washed with 1× DBPS (Dulbecco’s Phosphate Buffered Saline) (Ca^2+^/Mg^2+^ free, Gibco via ThermoFisher Scientific, Brisbane, QLD, Australia, #14190144) before being suspended with 0.05% Trypsin EDTA (Gibco, AUS, #25300054). 9LGS cells were harvested via this passaging method and counted and seeded for monolayers at 100% confluence into T12.5 cm^2^ flasks (Corning Incorporated, Corning, NY, USA, #353107) or 1 cm^2^ (well area) microchamber slide (Ibidi via DKSH Australia, Sydney, NSW, Australia, #80827) wells. Cell density for both vessels was set for 1 × 10^5^ cells/cm^2^ at confluence.

### 2.2. Halogenated Pyrimidine Preparation

Iododeoxyuridine (5-Iodo-2′-deoxyuridine) (IUdR) was obtained from Sigma-Aldrich as powder (≥99% (HPLC)) (via Merck Life Science, Melbourne, VIC, Australia, #I7125) and diluted in Hank’s Balanced Salt Solution (HBSS) (no phenyl red) (Gibco via ThermoFisher Scientific, Brisbane, QLD, Australia, #14175103) for a stock solution at 1.6 mg/mL. Stock solutions were prepared fresh and stored in a freezer at −19 °C for the duration of this study. An intermediate dilution of 100 μmol/L was produced in c-DMEM just prior to treatment. These dilutions were added to samples to obtain an optimal concentration of 10 μmol/L IUdR two doubling times prior to cells reaching 100% confluence (which was also the time of cell irradiation).

### 2.3. Chemotherapeutic Drug Preparation

Methotrexate (MTX) was obtained from Sigma-Aldrich (via Merck, Melbourne, VIC, Australia, #M8407). A dilution of 2 mg/mL of MTX was made with 2 mol/L (2 M) NaOH (Sigma-Aldrich via Merck, AUS, #S5881) in HBSS buffer (with phenyl red) (Gibco via ThermoFisher Scientific, Brisbane, QLD, Australia, #24020117) for a stock solution at pH 9.5. Stock solutions were prepared fresh and stored in a freezer at −19 °C for the duration of this study. An intermediate dilution of 0.1 μmol/L was produced in HBSS just prior to treatment. These dilutions were added to samples to produce a concentration of 0.01 μmol/L MTX two doubling times prior to 9LGS cells reaching 100% confluence.

### 2.4. Addition of Radiosensitiser Combinations

For combinations of IUdR and MTX (IUdR+MTX), both drugs were added at the same time the day after cell seeding, with two doubling times’ incubation at 37 °C and 5% CO_2_ (*v*/*v*) before irradiation.

### 2.5. Conventional Cell Irradiation Setup

Irradiation by conventional broadbeam (CBB) X-rays was performed at Prince of Wales Hospital, Randwick, NSW, Australia. This process followed the irradiation protocols of Oktaria et al. and Engels et al. Using a Nucletron Oldelft Therapax DXT 300 Series 3 Orthovoltage unit (Nucletron B.V., Veenendaal, The Netherlands), a 150 kVp kilovoltage peak energy (66 keV mean energy) was chosen to target the maximum mass energy absorption of thulium and iodine relative to water, demonstrated in our previous work [10,30,31,39]. Dosimetry was performed following the AAPM TG 61 protocol [43].

A clonogenic assay was performed for CBB irradiation at doses of 1, 2 5, and 8 Gy and contrasted with treated cells without radiation (0 Gy dose point). Doses of 5 Gy were used for microslide irradiation for γH2AX imaging. A monolayer of confluent 9LGS cells was used in T12.5 cm^2^ flasks for irradiation, all under 6 mm of complete DMEM culture medium. All flasks were irradiated horizontally, with a source-to-flask distance of 50 cm in full scatter conditions, including solid-water-adjacent and below the flasks. X-ray beam current was 20 mA and peak voltage was 150 kVp. Inherent tube filtration was 3 mm Be, and additional filtration was 0.35 mm of copper and 1.5 mm of aluminium (resulting in an HVL of 0.68 mm Cu). Dose rate was 0.75 Gy min^−1^ (0.0125 Gy/s) at the flask entrance [10,30,31].

### 2.6. Synchrotron Radiation Beam Configurations

Irradiation of cell samples was conducted in the Imaging and Medical Beamline (IMBL) of the Australian Synchrotron, Melbourne, Australia, using the dynamic option of IMBL’s hutch 2B. Doses of 2 and 5 Gy were used for SBB irradiations for the clonogenic assay in T12.5 cm^2^ flasks. Doses of 5 Gy were used for microslide irradiation for γH2AX imaging.

The synchrotron wiggler field was chosen as 2 T, with a Cu/Al filtration (71.4 keV mean energy to align the CBB energy and optimal absorption for the high-Z radiosensitisers) for SBB, which is comparable energy to CBB fields yet at several hundred times higher dose rates. The Cu/Al filtration used 1.41 mm of Cu and 2.82 mm of Al to produce a 71.4 keV mean energy for the SBB field. The intrinsic dose rate at 24 mm depth in solid water was 74.4 Gy/s.

The horizontal field size of 10 mm used for SBB necessitated the use of four columns and 6 cm vertical translation to cover the entire surface of T12.5 cm^2^ flasks and imaging microslides. Cell monolayers were irradiated in vertical position, with flasks fully filled with HBSS buffer (with phenyl red) (Gibco via ThermoFisher Scientific, Brisbane, QLD, Australia, #24020117), resulting in a 24 mm cell depth. All technical data and complete details for beam configuration parameters in vitro, including all dosimetry methods, can be found in our previous works [19,39,44] and further in Dipuglia et al. [45].

### 2.7. Clonogenic Cell Survival Assay

Clonogenic assays were performed for each treatment type to assess long-term cell survival [10,39,46,47]. Following treatment, 9LGS cells were subcultured and plated at low density in 10 cm diameter Petri dishes (Corning Primaria™ 100 mm Cell Culture [petri] Dishes, Corning Incorporated, Corning, NY, USA, #353803). After 15 doubling times of incubation, cells were rinsed with PBS (with Ca^2+^/Mg^2+^ salts, Gibco via ThermoFisher Scientific, Brisbane, QLD, Australia, #14040), fixed with 70% ethanol (*v*/*v*), and stained using crystal violet solution (Sigma Aldrich via Merck Life Science, Melbourne, VIC, Australia, #Ht90132) diluted 1:3 in 70% ethanol (*v*/*v*). Colonies with less than 50 cells were discounted, and plates with less than 50 colonies or more than 300 were discounted.

The plating efficiency (PE) was then determined as the ratio of the number of surviving colonies in a plate compared with the initial number of cells seeded. The surviving fraction (SF) was calculated as the ratio of the PE values for a treatment over that of the non-irradiated control (to obtain a percentage survival out of 100%).

### 2.8. Confocal Microscopy for γH2AX Imaging

DNA DSBs were imaged by confocal microscopy using γ-H2AX, a well-established DSB biomarker [48,49]. Microscopy was performed for a monolayer of 9LGS cultured for a confluence of 100,000 cells in the wells of an 8-well microchamber slide. One well was seeded for each treatment type, and at a confluence of 100%, cells were irradiated in accordance with the methods outlined in Section 2.5 and Section 2.6. The following protocol replicates our previous use of this assay [39,44].

20 min after irradiation, the cells were washed twice with 300 µL of ice-cold DPBS per well before being fixed with 300 µL of ice-cold 100% methanol per well for 20 min on ice. Wells were then washed three times each with 300 µL of cold DBPS, where for each wash the chambers were rocked at room temperature for 5 min. The chambers were then treated twice with a blocking solution of 3% bovine serum albumin (BSA) (Sigma-Aldrich via Merck Life Science, Melbourne, VIC, Australia, #A9418) in DBPS, with 15 min of rocking for each wash at room temperature. A primary antibody (Mouse anti-phospho-Histone H2A.X (Ser139), clone JBW301, supplied by Merck Millipore, Merck Life Science, Melbourne, VIC, Australia, #05-636) was added 1:500 in 1% BSA-DPBS for a concentration of 2 µg/mL to the cells, which were incubated for 2 h at room temperature.

Following incubation, the cells were washed three times at room temperature with BSA-DPBS and 5 min of washing per wash. A secondary antibody (goat anti-Mouse IgG1 Cross-Absorbed, Alexa Fluor 488, supplied by Invitrogen, Merck Life Science, Melbourne, VIC, Australia, #A21121) was added 1:500 in 1% BSA DPBS for a concentration of 4 µg/mL to the cells and incubated at room temperature in darkness for 1 h. Finally, cells were again washed twice with 300 µL of DBPS before 100 µL of DBPS was added to each well. An amount of 2 µL of 1 mg/mL Hoechst 33342 (Sigma-Aldrich via Merck Life Science, Melbourne, VIC, Australia, #14533) was then added to each well for 20 min at room temperature before cells were imaged with a Leica TCS SP8 confocal microscope with a 93× glycerol objective at room temperature.

The confocal microscope used a laser with a 488 nm excitation with a detection range for the Alex Fluor 488 fluorophore (FITC) and another 405 nm laser providing the range for the Hoechst 33342 nuclear counterstain (DAPI). Detection ranges were set to a minimum of 10 nm above the excitation wavelengths for each laser. A 2 × 2 tile scan with a z-stack of 10 slices was taken per image. These images were then analysed via the Lecia LasX Application Suite (v. 3.0.11.20652, Leica, Microsystems, Wetzlar, Germany), ImageJ (v 1.53k; NIH, Bethesda, MD, USA) [50], and Microsoft Excel (v. 2016; Redmond, WA, USA).

### 2.9. Image Processing and Analysis

ImageJ (v 1.53k) [50] was used to process images to quantify DSBs observed in a γH2AX image (which are represented by γH2AX foci). A quantitative analysis of γH2AX foci was used as the key indicator of DNA damage due to the high sensitivity of this method [48,49]. For this study, the foci factor (FF) method was used to account for variations in individual γH2AX foci. This method follows our previous work establishing this method in Valceski et al. [44].

An FF value offers a metric representing the average number of DSBs per cell nuclei. The FF value is determined for each individual image as the raw integrated density (the total sum of pixel intensity values in a focus) summed up across all foci in that image, divided by the number of cells counted (analytically via ImageJ) in the image. Following this method, the DSB enhancement ratio (DSBER) discussed in Section 3 was determined as the ratio of foci factors of a treatment sample to the untreated, 0 Gy control (Equation (1)).

(1) $$\frac{FF_{treatment}}{FF_{control}}=\frac{DSB_{treatment}}{DSB_{control}}=DSBER$$

This DSBER value was used as the final quantification of all confocal images using the γH2AX assay in this study and represents a quantification of the enhancement in DSB induction in a sample. The DSBER values of at least six images were averaged for each sample.

Additionally, qualitative analysis was also performed using ImageJ and the Lecia LasX Application Suite. Image processing and qualitative observation at these high resolutions permitted a manual tally of biological features and hallmarks of cell death, including apoptotic and necrotic pathways common following irradiation [51,52,53,54]. Once counted manually, a full cell count of that image permitted the percentage of the population exhibiting markers and/or undergoing some cell death pathway to be calculated. The average percentage (of at least three images per sample) was calculated and compared. Accordingly, an image panel, overlaying fluorescent channels with the optical bright field channel showing the cell morphology, was produced to highlight specific biological features observed and tallied.

### 2.10. Statistical Analyses

All error bars were calculated as standard error using 2 standard deviations of the mean (at the 95% confidence interval) divided by the square root of the number of images used (i.e., FF values being averaged) or clonogenic plates or samples counted. For all samples tested, at least 4 replicates across independent repeats were averaged for each sample.

A Student’s *t*-test was used to compare samples for statistical significance, with the unpaired heteroscedastic *t*-test for all independent samples. One-tailed *t*-tests were used when comparing to untreated controls, as the increase was the primary interest, while all other cases used a two-tailed *t*-test. The *p* values for each statistical test are presented in the corresponding figure legend.

## 3. Results

### 3.1. SBB Reduces 9LGS Clonogenic Survival Compared to CBB

**Figure 1:**
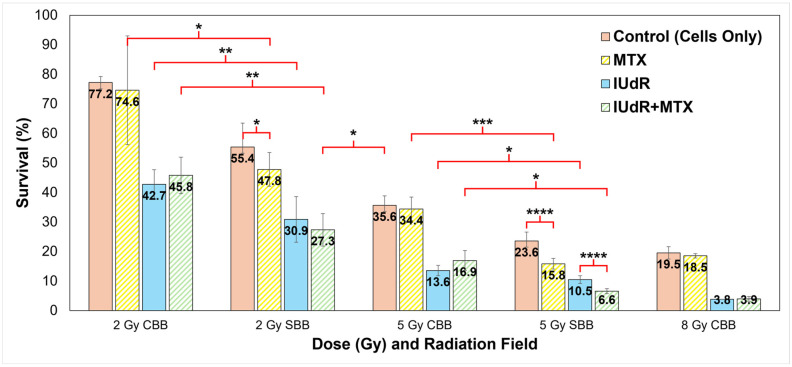
Clonogenic survival for all treatment combinations in all fields and at all dose rates. Shown are all radiation fields and radiosensitisers used to treat 9LGS cells using a single RT fraction. Doses of 2 Gy and 5 Gy are compared for each of CDR CBB orthovoltage X-ray fields at 150 kVp (66 keV mean energy) at 0.0125 Gy/s and UHDR SBB fields using a 2T Cu/Al filter (71 keV mean) at 74.1 Gy/s. An 8 Gy CBB field is shown for comparison. The 5 Gy data are sourced from our previous work in Valceski et al. [39], with new 2 Gy and 8 Gy compared in this study. Cells-only controls (solid red/pink columns; radiation-only; no radiosensitisers) are compared with methotrexate (MTX; yellow striped columns), iododeoxyuridine (IUdR; solid blue columns), and IUdR+MTX combinations (green striped columns). All surviving fractions are normalised to the non-irradiated control (no radiation, which represents 100% survival). For statistics, an average of *n* ≥ 6 replicates are used for each data point; error bars represent standard error of the mean at the 95% confidence interval; and for *p* values: (*) = *p* < 0.1, (**) = *p* < 0.05, (***) = *p* < 0.01, (****) = *p* < 0.001.

Figure 1 clearly demonstrates a significant clonogenic survival reduction when doses were delivered with SBB compared with CBB X-rays. Synchrotron radiation produced a 28% reduction in 9LGS clonogenic survival at 2 Gy and a comparable 34% fall at 5 Gy relative to the conventional orthovoltage. Indeed, the 5 Gy SBB survival is comparable to and well within statistical error of the 8 Gy CBB fraction despite the conventional X-rays delivering a much higher radiation dose.

These significant reductions in 9LGS cell survival seen in Figure 1 with UHDR SBB fields are observed despite the dose rate being thousands of times higher than that of the corresponding CDR CBB fields. This indicates that radiation dose rate alone was able to potentially improve treatment efficacy at both 2 Gy and 5 Gy (Figure 1). This occurred even without radiosensitisers like IUdR and MTX present.

### 3.2. High-Z DNA-Localised Radiosensitisers Enhance Conventional Radiotherapy

Intrinsic toxicity of the radiosensitisers was first assessed without radiation (0 Gy). Clonogenic survival at 0 Gy was found to be (88.3 ± 17.1)% for MTX and (60.6 ± 11.7)% for IUdR, while survival was (55.6 ± 13.0)% for IUdR+MTX. Results in Figure 1 are normalised to these survival values to demonstrate the effect of radiation enhancement specifically. It is clear that when radiosensitiser agents are used to pre-treat 9LGS cells, the effect of the radiation is overall greatly enhanced. Notably, IUdR treatment prior to CBB irradiation delivery significantly enhances radiation effects with, for example, a 62% decrease in survival at 5 Gy compared with radiation alone.

Nevertheless, Figure 1 also demonstrates no significant change in survival when 9LGS cells were treated with MTX and CBB X-rays. The addition of MTX, singularly or in combination with IUdR, did not change cell survival compared to CBB. This correlated with a similar result found by Oktaria et al., in which no dose enhancement was observed with 9LGS cells when using 0.01 μmol/L MTX and 125 kVp conventional X-rays [31].

### 3.3. Radiosensitiser Combinations Are Highly Synergistic in High Dose Rate Synchrotron Fields

By contrast, Figure 1 tells a completely different story with MTX enhancement in SBB fields. As previously revealed in Figure 1, the UHDR synchrotron X-rays delivered a dose rate effect such that greater 9LGS cancer killing occurred with UHDR radiation. Accordingly, the greatest synergistic effect was then observed when 9LGS cells were pre-treated with MTX, which is known to impede cell growth by preventing DNA replication and repair through the arrest of the folate cycle [31,33].

Figure 1 clearly demonstrates that SBB with MTX produced significant enhancement, whereby 9LGS survival fell by 56% relative to CBB with MTX at 5 Gy, far greater than the 34% reduction seen with radiation alone. We theorise that this may result from the disruption of DNA repair pathways [33], whereby the UHDR X-rays may overwhelm 9LGS cells with too many DSBs to repair, leading to cell death [55,56]. MTX therefore reduced 9LGS survival by an additional 22% when combined with SBB, demonstrating a clear synergistic or supra-additive effect with UHDR.

This synergy was amplified when MTX was combined with a high-Z radiosensitiser, where a combination of IUdR+MTX induced more cell killing at 5 Gy than with high-Z IUdR alone (Figure 1). As this was not observed at 2 Gy, this indicated a potential dose dependence of combinational synergy. IUdR+MTX significantly reduced 9LGS cell survival with SBB at 2 Gy, with cell survival now lower than 5 Gy CBB radiation-only with UHDR X-rays despite less than half the dose (Figure 1). With 5 Gy SBB, the fall in survival was 82% lower for IUdR+MTX compared to 5 Gy CBB radiation alone, amounting to 5.4 times more cancer killing than a typical conventional fraction at the same dose. This treatment yielded the greatest 9LGS cell killing found in this study and provided the same level of cancer cell killing as a conventional 8 Gy X-ray fraction (Figure 1) but with a notably lower dose per fraction (only three-fifths).

When compared to the 5 Gy SBB control to account for dose rate effects, the enhancement seen with these combinations was greater than any treatment individually. With 5 Gy SBB, Figure 1 demonstrates a 72% lower survival with IUdR+MTX (6.6%) than radiation alone (23.6%). These results confirmed that high-Z radiosensitisers combined with a chemotherapeutic agent such as MTX can have a supra-additive effect with UHDR radiation fields like SBB.

### 3.4. High-Z Materials Enhance DNA Damage Following Irradiation

**Figure 2:**
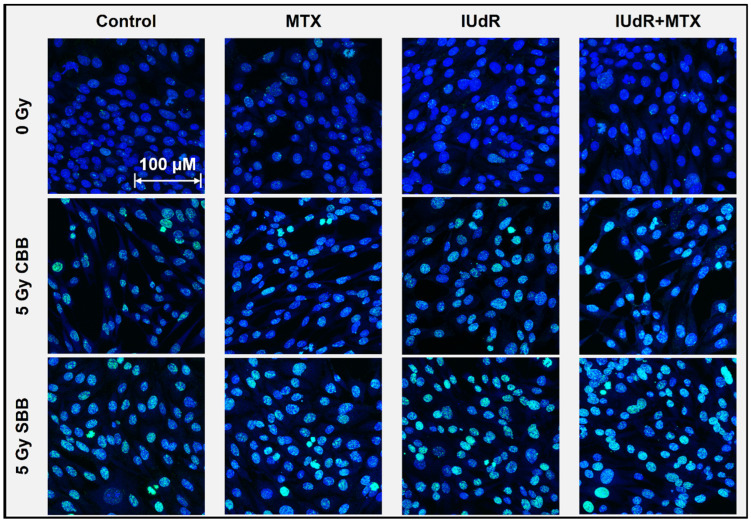
Double-strand DNA breaks following 9LGS cell irradiation. This γH2AX confocal microscopy image panel shows representative 93× resolution images of 9LGS cells as the maximum projection of 10-slice Z-stacks. DSBs are represented by green γH2AX foci (FITC) overlayed on a Hoechst 33342 (DAPI) nuclear counterstain (blue). Radiosensitiser treatments are displayed in columns across the panel (with cells-only controls for comparison; leftmost column) and show DSB changes 20 min after irradiation began for all cases in 9LGS cells. Radiation treatments change down the rows and show the 0 Gy case (top row) compared with 5 Gy CBB X-rays (middle row) and 5 Gy SBB fields (bottom row). Cells were fixed immediately at 20 min post-irradiation. The scale bar provided applies to all images in the panel.

To unveil the mechanisms behind the results presented in Figure 1, both γH2AX immunofluorescence staining and nuclear counterstaining of adherent 9LGS cells was performed shortly after irradiation. High-resolution images for all pre-treatments with and without radiation are presented in Figure 2.

Figure 2 highlights minor increases in DSB incidence with IUdR present, even without radiation (0 Gy). This may be expected, as Kinsella previously found IUdR to induce DNA damage signalling in cells [57]. The greater incidence with conventional treatments is found following CBB X-ray exposure. While the radiation alone demonstrates visible increases, IUdR and IUdR+MTX show the greatest enhancement in DSB incidence, correlating well with Figure 1 results showing increased cancer killing due to this greater DNA damage. Accordingly, MTX is not significantly different from radiation alone and shows no synergy with CBB (as observed in Figure 1).

The greatest increase in DNA damage is found when 9LGS cells are exposed to UHDR SBB fields alone. This explains some of the underlying mechanisms behind the increased cell killing seen with the UHDR radiation. While UHDR radiation is typically associated with sparing, our results in Figure 2 indicate increased DNA damage and hence cell killing, as observed in Figure 1. There are more, larger, and brighter γH2AX foci present, suggesting that fast dose delivery results in significant DNA damage. It is also important to note that these images are taken in the 20 min immediately following irradiation, so longer-term repair has yet to take place.

Accordingly, the greatest DSB enhancement is observed in Figure 2 with IUdR+MTX. The presence of DNA-localised, high-Z IUdR naturally increased DNA damage following irradiation, hence resulting in reduced survival in Figure 1. The synergistic combination of IUdR+MTX with UHDR fields appears to have the brightest, largest, and most foci in 9LGS cells (Figure 2), indicating the greatest propensity for cell death due to such overwhelming damage so quickly.

### 3.5. High Dose Rate Fields Increase Incidence of Cell Death Following Treatment

**Figure 3:**
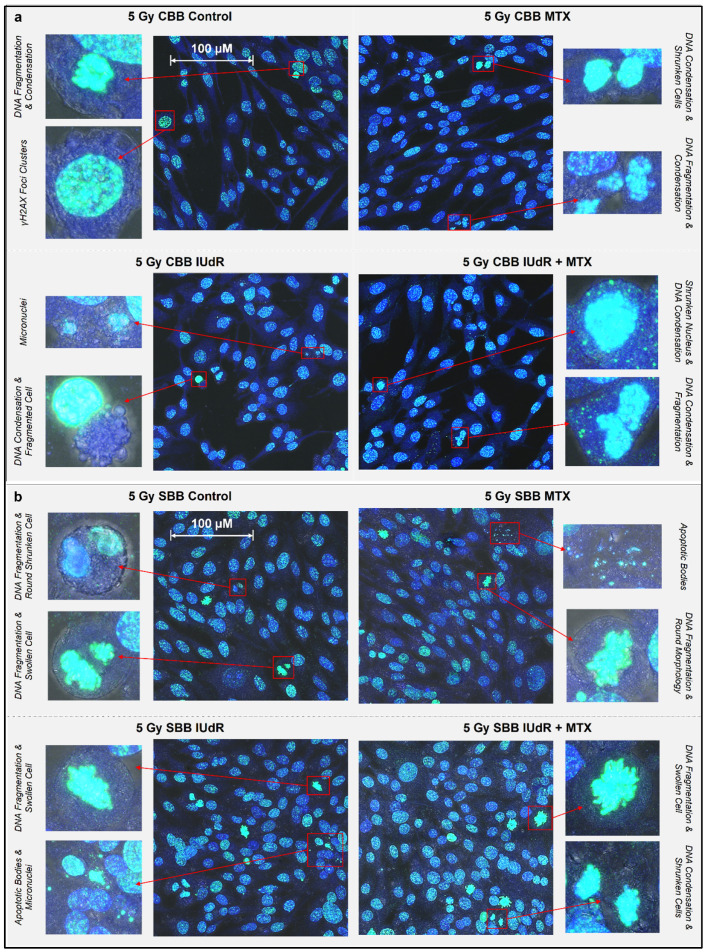
Morphological features and radiobiological cell response following irradiation. γH2AX images (maximum projections of 10-slice Z-stacks) were taken via 93× resolution confocal microscopy showing nuclear and morphological features of 9LGS cells following treatment. All images overlay γH2AX foci representing DSBs (green—FITC), Hoechst 33342 nuclear counterstain (blue—DAPI), and optical bright field. One representative image is shown for each of the cells-only control and the MTX, IUdR, and IUdR+MTX treatments, each following irradiation with either a CBB field (**a**) at 0.0125 Gy/s or SBB field (**b**) at 74.1 Gy/s. Two zoomed-in close-ups of cell morphological features are shown beside each image. Cells were fixed immediately at 20 min post-irradiation. The scale bars provided apply to all images in that panel.

**Figure 4:**
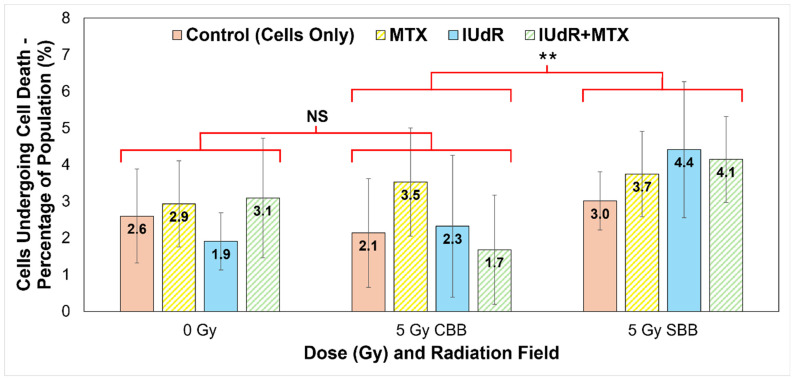
Tally of cells exhibiting morphological signs of cell death as a percentage of population. Nuclear morphological changes were used as the primary method of tally, including signs of chromatin condensation, DNA fragmentation, and nuclear membrane distribution and leakage. Cytoplasmic morphological changes include cell shrinkage and swelling and membrane ruptures or the presence of apoptotic bodies. Manual tally was counted for all γH2AX confocal microscope 93× resolution image sets represented in Figure 2 (cells fixed at 20 min post-irradiation), i.e., cells-only control (solid red/pink), MTX (yellow stripes), IUdR (solid blue), and IUdR+MTX (green stripes), for 0 Gy, 5 Gy CBB fields, and 5 Gy SBB fields. The total number of cells in each image was counted, and values represent the average percentage of cells in each population manually tallied as exhibiting signs of cell death (represented in Figure 3). For statistics, an average of *n* ≥ 4 replicates are used for each data point; error bars represent standard error of the mean at the 95% confidence interval; and for *p* values: (**) = *p* < 0.05 and NS = not significant. For statistical groups, the average of the four treatments are compared using a two-tailed heteroscedastic Student’s *t*-test (i.e., the four data points for one radiation field are compared with the four data points in another to demonstrate, regardless of treatment agent used, if changes are significant across fields).

Increased cell death following radiosensitiser-enhanced UHDR radiation is shown in Figure 3 and Figure 4. The significant DSB levels observed in Figure 2 demonstrate that an abundance of damage can also induce biological signs of cells entering a death pathway in their early stages within the immediate 20 min after irradiation. This is shown in Figure 3, where DNA fragmentation is seen commonly across treatment variations, as are DNA condensation, micronuclei, apoptotic bodies, and morphological changes including shrinkage or swelling.

With apoptosis, the programmed death of the cell, morphological observations in the early stages of this pathway are cell shrinkage and pyknosis (condensation of nuclear material such as a chromatin) as the most characteristic features [51,52]. The shrunken cell membrane appears as round with dense chromatin fragments during apoptosis and can later lead to karyolysis (dissolution of the chromatin) and the disintegration of the cell into apoptotic bodes [51,52].

By contrast, necrosis, the uncontrolled death of the cell, often following cell injury, sees cell swelling and karyorrhexis (destructive fragmentation of the nucleus that distributes unevenly throughout the cytoplasm) in the early stages [51,52]. It is clear in Figure 3, given the common examples of nuclear fragmentation and chromatin condensation across treatment types, that apoptosis may be a possible and common cell death mechanism following irradiation. Examples of necrosis were also observed in several samples across treatments. These findings are expected as these common cell death pathways, as well as mitotic catastrophe and senescence but notably apoptosis, are typical following ionising-radiation-induced cell death, including in GBM [53,54]. Figure 3 demonstrates that these death pathways were present across treatments regardless of radiosensitiser or pre-treatment of 9LGS cells with MTX.

While significant changes in cell death incidence may take days to be visible and the images in Figure 3 were taken of 9LGS cells fixed at 20 min post-irradiation, apoptosis can also be executed within approximately 10 min, although the initial triggers may have been hours or days earlier [51,52,58,59,60]. Figure 2 highlights that both CBB and SBB radiation fields resulted in the initiation of some cell death pathways, as evidenced by the hallmarks of apoptosis, necrosis, and more. While further study is needed to verify which cell death pathways have been triggered and accurately quantify the extent of increased cell death, Figure 3 still indicates that some cell death was potentially already underway in the short period after irradiation.

Accordingly, a manual count of the image sets represented in Figure 3 yielded the results presented in Figure 4. Manual count of the number of cells exhibiting known markers of cell death followed by a full population cell count of the image allows the proportion of cells likely undergoing some form of cell death to be quantified. Figure 4 then clearly highlights the effect of the UHDR radiation on 9LGS cells in vitro.

9LGS cells are found in Figure 4 to be undergoing a statistically significantly increased level of cell death in the short time after irradiation with UHDR SBB fields. The average proportion of potentially dying cells observed for SBB fields across treatments and regardless of radiosensitiser used is found to be (3.83 ± 0.53)%. This figure is determined by ignoring differences between treatments and simply calculating the average and uncertainty of all data points at that dose rate (SBB or CBB). Accordingly, the average value in Figure 4 for the CDR X-rays is (2.42 ± 0.68)%, which is significantly lower than the SBB value by 37% and outside of experimental error (*p* = 0.032).

**Figure 5:**
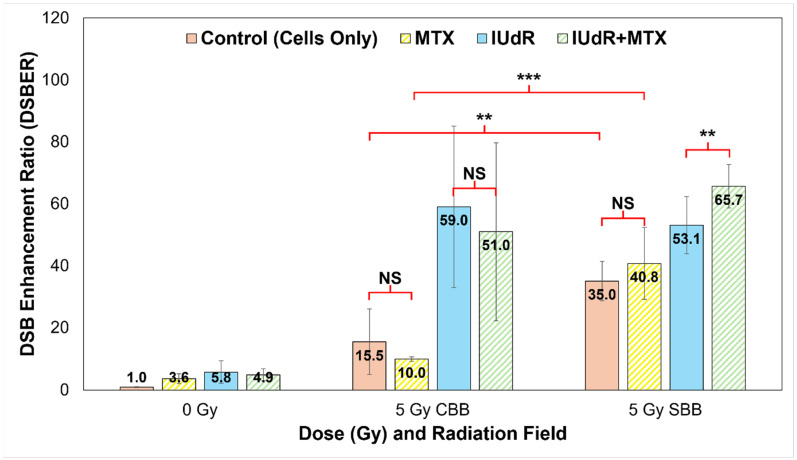
Quantification of γH2AX DNA DSBs. We use a quantitative analysis of 93× resolution confocal images (represented in Figure 2; all cells fixed at 20 min post-irradiation) to quantify DNA damage in treatments. Cells-only controls (solid red/pink) are compared with MTX (yellow stripes), IUdR (solid blue), and IUdR+MTX (green stripes) for 0 Gy, 5 Gy CBB fields, and 5 Gy SBB fields. Results yielded DSB enhancement ratios (DSBER) relative to the 0 Gy cells-only (untreated) control sample, expressed in terms of the ratio of FF of each treatment normalised to the FF of the untreated 0 Gy control. The 5 Gy data are sourced from our previous work in Valceski et al. [39], with new 0 Gy compared in this study. For statistics, an average of *n* ≥ 4 replicate images are analysed for each data point; error bars represent standard error of the mean at the 95% confidence interval; and for *p* values: (**) = *p* < 0.05, (***) = *p* < 0.01 and NS = not significant.

Accordingly, Figure 3 and Figure 4 correlate strongly with Figure 2 and Figure 5, where increased DSB incidence is observed with UHDR SBB fields, indicating that the UHDR X-rays induce significant levels of DNA damage that can in turn trigger increased cell death. Figure 4 clearly demonstrates that UHDR synchrotron X-rays induced significantly more 9LGS cell death following irradiation, further correlating well with results in Figure 1 demonstrating increased cell killing with SBB fields. A causal link is indicated that UHDR fields appear to induce significant DSBs following irradiation (Figure 2), which in turn triggers increased cancer cell death shortly thereafter (Figure 3 and Figure 4), resulting in significantly reduced long-term survival (Figure 1). This is verified and further supported in Figure 5, which indicates the greatest cumulative effect.

### 3.6. Synergistic Radiosensitisers Increase DNA DSBs in High Dose Rate Fields

Using our foci factor method described by Equation (1), a full quantification of the γH2AX images represented in Figure 2 was performed. This produced DSB enhancement ratios to determine the proportion of increased DNA damage in response to treatments, both at different dose rates and with different radiosensitisers. These results are shown in Figure 5 and clearly demonstrate the effect of both high-Z radiosensitisers and UHDR X-rays.

Figure 5 confirms the significant increases in DNA damage visible in Figure 2 with high-Z IUdR present. Correlating well with Figure 1, IUdR alone induced significantly more DSBs with both CDR fields and UHDR SBB X-rays, likely resulting from a combination of the low energy, high-Z photoelectron cascades as well the halogen’s proximity to DNA. Accordingly, the lack of synergy with MTX is clearly visible with CBB, resulting in no significant differences compared with IUdR or X-rays alone. This may result from potential radioresistance of 9LGS further enhanced by MTX shifting 9LGS in the S (synthesis) phase of its cell cycle, which is known to be radioresistant [31,61,62,63], hence resulting in fewer DSBs in Figure 2 and Figure 5.

However, the opposite is true with SBB fields. The UHDR X-rays clearly induce significantly more DSBs in 9LGS cells than CDR radiation in Figure 5, with nearly double the level of damage. This correlates well with Figure 1 where increased long-term cancer killing is observed with UHDR X-rays at both lower and higher doses compared with CDR. Accordingly, MTX synergy is then observed as well, where SBB fields induce nearly four times more DSBs. This suggests that potential radioresistance issues may have been overcome and hence correlates with the synergistically enhanced 9LGS cell kill in Figure 1. Despite this, there does not appear to be significantly more DSBs with SBB MTX treatment than with UHDR X-rays alone, indicating some other mechanisms may be at play to result in the synergistic improvement in long-term cancer killing in Figure 1.

Regardless, this consequently resulted in a significant increase in DSBs with IUdR+MTX, which induced the highest levels of DNA damage following UHDR SBB irradiation. As such, this links with the high levels of increased cell death with this treatment regimen shown in Figure 4 and hence likely resulted in the significant long-term cancer killing seen in Figure 1, where IUdR+MTX with UHDR SBB fields resulted in 5.4 times more cancer killing than a conventional 5 Gy radiation-only fraction alone. Evidently, this overwhelming level of DNA damage may be the underlying mechanism for the exceptional synergy observed with this ultra-fast combinational radiotherapy. This bodes well for future treatment potential given that UHDR radiotherapies are typically associated with normal tissue sparing, while this study demonstrated that a highly synergistic multi-modal approach can provide a new paradigm for improved tumour control as well.

## 4. Discussion

Our results follow our previous work in Valceski et al. [39], which compared the use of radiosensitisers in therapeutic synchrotron fields collimated into microbeams [20]. While the previous study focused on synchrotron microbeam fields enhanced by high-Z nanoparticle radiosensitisers [64,65,66], high-Z IUdR halogenated pyrimidine and MTX chemotherapeutic drugs were explored primarily for comparison. In this study, In this study, our focus is on the radiosensitiser drugs instead and the effects of ultra-high dose rate fields more generally. Similar datasets are utilised and re-analysed to explore the effects of dose rate and its synergy with DNA-localised high-Z agents and MTX chemotherapeutics. Additional data are also provided, and new comparisons are made complete with a thorough statistical analysis. Notably, a clonogenic cell survival assay is used to compare efficacy enhancements, whilst mechanistic explanations are provided via a comprehensive biological approach using high-resolution γH2AX confocal microscopy.

Accordingly, this study demonstrates significant enhancement in treatment of 9LGS cells with supra-additive IUdR+MTX combinations when combined with synchrotron broadbeam X-rays. This is despite no additive effects being observed with IUdR+MTX when 150 kVp CBB radiation is used (Figure 1). However, an additive effect with bromine-based pyrimidine BrUdR (10 μmol/L), rather than 10 μmol/L IUdR, at 125 kVp CBB X-rays was previously observed [31]. McDonald et al. found a similar result, whereby 10 μmol/L BrUdR was found to be synergistic in combination with chemotherapeutic 5-FU using 125 kVp X-rays [30]. It is possible that the energy used may affect the result in Figure 1 as the radiation beam energy and dose rates differed, as did the element used (bromine in BrUdR compared with iodine here). It is also possible the 0.01 μmol/L concentration of MTX used may be too low to induce an additive effect under conventional conditions. This also demonstrates that enhancement with CBB X-rays observed in Figure 1 is likely driven by the high-Z radiosensitiser rather than chemotherapeutic drugs like MTX.

Regardless, MTX drugs still demonstrate significant synergy with IUdR and UHDR X-rays. IUdR+MTX reduced 9LGS survival with SBB radiation by a factor of 5.4 compared to conventional 5 Gy dose fractions (Figure 1), demonstrating the highly synergistic effects of combining high-Z radiosensitisers with anti-cancer drugs like MTX that hinder cell growth and repair. This ultra-fast combinational RT significantly demonstrated significant efficacy using this multi-modal approach, whereby a single 5 Gy fraction reduced 9LGS cancer survival by nearly 95% and exhibited cancer control comparable to an 8 Gy fraction (Figure 1).

The underlying mechanisms revealed in Figure 2, Figure 3, Figure 4 and Figure 5 indicate that this dose enhancement resulted from overwhelming double-strand DNA damage, which in turn resulted in significantly increased cell death prevalence shortly following irradiation. This in turn likely induced the long-term improvement in cancer killing shown in Figure 1, notably with high-Z IUdR drugs. The presence of DNA-incorporated high-Z material in the DNA may have increased the potential for nuclear DNA damage. A potential driver of high-Z enhancement and cancer cell death may also be the increased incidence of DSBs resulting from secondary electron emission from radiosensitisers like IUdR [10,31]. Similar results have been observed in previous studies, where high-Z nanoparticles were found to contribute to reduced cell survival, with this suggested as being potentially due to DNA damage such as DSBs [39,67]. These results further supported, in combination and singularly, the benefit of DNA-localised agents compared to cytoplasmic distributions as seen in our previous work focusing on the use of high-Z nanoparticles with UHDR radiation, notably synchrotron microbeams [39].

The use of UHDR SBB X-rays demonstrated a significant dose rate effect that allowed SBB radiation alone to greatly reduce 9LGS survival even further when compared to the CBB counterpart at the same doses (Figure 1). These significant increases in cancer killing for UHDR fields were observed despite the SBB intrinsic dose rate being approximately 6000 times greater than the corresponding conventional orthovoltage at the same depth in water. The SBB fields were also delivered at a higher dose rate than 40 Gy/s, which has recently been considered the threshold for normal-tissue-sparing FLASH effects [16,18]. Despite dose rate effects typically being related to normal tissue sparing and protection, this study found a significant increase in cancer killing with UHDR X-rays alone, suggesting a dual benefit of UHDR RT, as noted by Engels et al. [19]. Previous work also at the Australian Synchrotron also found the same UHDR effect, with a 2 Gy SBB dose fraction at an equivalent dose rate of 50 Gy/s also reducing survival of 9LGS by 42% with radiation alone [68].

Exposure of 9LGS to MTX prior to irradiation further reduced clonogenic survival, demonstrating a clear supra-additive effect with the anti-cancer drug and UHDR radiation (Figure 1). This further highlights a potential additional attribute of dose rate effects, where the UHDR alone produces significantly more damage to cancer cells with radiosensitisers present, which contrasts with traditional normal-tissue-sparing and protection attributes normally associated with UHDR and FLASH effects [16,18].

Despite this, Figure 5 reveals a fifteen-fold increase in DSBs 20 min after 5 Gy of CBB X-rays are used to treat 9LGS cells, supporting the qualitative results shown in Figure 2 and correlating with reduced survival with UHDR X-rays shown in Figure 1. MTX, however, saw a reduction in DSBs, which may explain why it did not reduce 9LGS survival (Figure 1) with CBB. Oktaria et al., having found a similar result for 9LGS cells treated with the same concentration of MTX (0.01 μmol/L), also observed by cell cycle analysis an increase in the population of cells in S phase following exposure to MTX [31]. With the inhibition of folate production in the cell, MTX can slow the cell cycle after cells transition from G1 to S phase, which therefore results in a sizeable part of the cell population gathering in S phase at the expense of G2/M [31,61].

It is well-known that cells have different radiosensitivity in different cell cycle phases, with cells in the late S phase being the most resistant and G2/M being the most sensitive [69], although this depends on the cell line [70]. 9LGS cells are likely to be radioresistant in S phase, suggesting upregulation and repair of DNA damage following irradiation [61,62,63]. The gathering of 9LGS cells in radioresistant S phase would explain the lack of enhancement provided by MTX in 9LGS, in both this study and the findings of Oktaria et al. [31], which is notable given that Figure 5 demonstrates only just over half the increase in DSB production with MTX compared to radiation-only. Ultimately, more study is needed to verify this empirically, but it does not take away from the clear enhancement in efficacy observed via synergy with UHDR SBB radiation.

Figure 2 and Figure 5 also show relative equivalence in DSB production following irradiation of high-Z IUdR-treated 9LGS cells with either SBB or CBB, despite reduced SBB survival in Figure 1. While γH2AX data only reveal DSBs, SSBs are also produced and are known to increase with UHDR synchrotron radiation [19]. SSBs can also convert into DSBs during cell replication if improperly repaired [54,71]. Inhibition of repair by MTX, or increased DNA damage due to secondary radiation from high-Z radiosensitisers like IUdR, may result in further DSBs (lethal events in clonogenic survival), as seen in Figure 2, Figure 3, Figure 4 and Figure 5.

While it may be expected that if significant numbers of SSBs are produced and converted, then these would be revealed by γH2AX, it should be noted that the data shown in Figure 2, Figure 3, Figure 4 and Figure 5 represent DSBs induced within 20 min of irradiation, while clonogenic survival in Figure 1 represents long-term effects over several weeks. As such, it is possible that the additional cancer-killing results from conversion of many irreparable SSBs (sublethal events) to DSBs (lethal events) over time. This may also explain why MTX synergistically increased 9LGS cancer killing with UHDR X-rays, shown in Figure 1, beyond SBB radiation alone yet saw no significant difference in DSB incidence shortly after irradiation, shown in Figure 5.

Whilst DNA damage has many pathways for repair, SSBs are commonly repaired quickly by base excision repair (BER), whilst more disastrous DSBs may be repaired by homologous repair (HR) or non-homologous end joining (NHEJ) pathways, which may take far longer [13,55,72]. Should repair of dominant SSBs in the short period following irradiation be inhibited or overwhelmed by DNA damage, it is possible that long-term conversion of SSBs into accumulating DSBs over time will occur. When cells are treated by growth inhibition drugs such as MTX, which is known to inhibit HR pathways used for DSB repairs [33], the probability of excessive DSBs resulting in cell death would further increase.

We propose the following theory for a mechanistic explanation of our UHDR results (notably, given that UHDR effects typically infer tissue protection [16,18] rather than the cancer killing seen in Figure 1). We propose there may be a significant number of additional SSBs induced in the short term with UHDR SBB fields that may result in lower cell survival in the long term (Figure 1). The high dose rates would deliver many X-ray photons very quickly and therefore induce significant DNA damage in a very short period (Figure 2 and Figure 5). The severity of damage could then overwhelm DNA repair capacity [13,54,55,71,72,73], leading to tumour cell death via pathways such as apoptosis or necrosis (Figure 3 and Figure 4) [51,52,53,54]. Any additional SSBs, which comprise most genetic initial lesions, could be converted to DSBs over time [54,71], further exceeding the DNA repair capacity of the cell though overwhelming damage, hence increasing the probability of cell death (Figure 3 and Figure 4) [13,55,56,71,72,74]. When radiosensitisers are used, the additional dose enhancement provided would further increase immediate DNA damage and therefore the likelihood of overwhelming 9LGS (Figure 2, Figure 3, Figure 4 and Figure 5).

This may explain the reduced cell survival (Figure 1) and increased DSB production (Figure 2 and Figure 5) resulting in increased cell death (Figure 3 and Figure 4) with all RT modalities when pre-treated with radiosensitiser agents, especially highly synergistic combinations like IUdR+MTX with UHDR SBB X-rays. Ultimately, while this may be a potential underlying mechanism to link our findings in this study, we note that additional investigation to empirically quantify SSB incidence and conduct time courses to track any potential DSB build-up and repair over time will be necessary to verify this hypothesis. Nonetheless, this study still highlights a significant novel effect with UHDR RT. This further verifies the potential for improving the efficacy of cancer treatment via ultra-fast, multi-modal radiosensitiser combinations like IUdR+MTX, notably so given that this highly synergistic approach killed nearly 95% of a notoriously treatment-resistant 9LGS cancer population in a single 5 Gy dose fraction.

Despite the known limitations of this study (including limited dose points, single-drug concentrations, one cell line, and a short time course for DNA damage and cell death studies), it still unveiled a significant increase in DNA damage and cell death following UHDR irradiation in combination with MTX and IUdR. Future studies are required to deepen our understanding of the underlying DNA damage and cell death mechanisms, including repair kinetics, that resulted in such significant increases in cancer cell killing.

## 5. Conclusions

This study has aided our understanding of the cancer treatment capabilities of UHDR radiotherapy and provided several novel and methodical approaches to its comprehensive radiobiological analysis. While UHDR radiation effects are typically associated with tissue protection, our investigation found a potential additional paradigm where improved tumour control via overwhelming initial damage may be possible. Future studies may consider deeper investigation of this extensive damage, including DNA damage repair over time. Particularly, significant cancer damage and control via localised radiosensitiser combinations enhanced supra-additive efficacy in these multi-modal cancer therapies. These ultra-fast, highly synergistic combinations of IUdR+MTX with UHDR X-rays were demonstrated to induce 5.4 times more cancer cell killing in a single fraction. This bodes well for future investigations of radiosensitiser-enhanced synchrotron radiotherapy both in the label and in future pre-clinical settings where a synergistic multi-modal may be the key to highly successful cancer treatment with minimal dose fractions.

## Data Availability

Data are available within this paper. The raw data supporting the conclusions of this article will be made available by the authors on request. All image analysis code functions are available via ImageJ from the National Institutes of Health, United States (https://imagej.net/ij/index.html, accessed on 16 May 2025). All computational functions for dataset analysis and calculations are available in Microsoft Excel from the Microsoft 365 software suite (https://www.microsoft.com/en-au/microsoft-365/excel, accessed on 16 May 2025). All were applied to image analysis of confocal microscopy images in accordance with the methods and protocols listed within this article.
